# Target detection enhances relational processing of background items: evidence from associative recognition paradigms and EEG

**DOI:** 10.1186/s40359-026-04717-w

**Published:** 2026-05-16

**Authors:** Peijie Chen, Yingfang Meng, Zejun Liu

**Affiliations:** 1 School of Psychology, Fujian Normal University, Fuzhou, 350100 China; 2 School of Psychology, Shanghai Normal University, Shanghai, China

**Keywords:** Attentional boost effect, Item-specific vs. relational processing, Associative memory, Familiarity, Recollection

## Abstract

**Background:**

The Attentional Boost Effect (ABE) refers to the enhancement of memory for concurrently presented background information when target detection is performed during encoding. However, there is still controversy regarding whether ABE-related manipulations can both enhance item-specific and relational processing. Given that resolving this issue can enhance our understanding of the generality of the ABE, we will validate this issue and explore its underlying neural mechanisms.

**Methods:**

The current study combined the ABE paradigm with the associative recognition paradigm and employed compound pairs and unrelated pairs as memory materials to manipulate the reliance on item-specific or relational information, using a 2 (detection stimulus type: target vs. distractor) × 2 (word pair: compound vs. unrelated) within-subject design. Furthermore, we conducted event-related potential (ERP) and time–frequency analyses (TFA) on participants' electrophysiological data during associative recognition to further explore the underlying neural mechanisms of the ABE.

**Results:**

The results showed that, behaviorally, participants remembered more target-paired pairs than distractor-paired pairs for both types of word pairs, demonstrating an ABE. In the ERP results, target word pairs evoked a significant FN400 effect, related to familiarity, whereas distractor word pairs did not evoke this effect. Furthermore, for compound pairs, target pairs evoked larger LPC effects than distractor pairs, which were related to recollection; however, for unrelated pairs, there was no difference in the LPC effect between target and distractor pairs. The TFA results showed that target word pairs induced lower gamma-band activity than distractor word pairs, reflecting familiarity processing.

**Conclusions:**

Taken together, target detection enhanced associative memory performance, indicating that ABE-related manipulations can both facilitate item-specific and relational processing. At the neural mechanism level, ABE-related manipulations enhanced the contribution of familiarity in associative recognition, and when the semantic association between items was stronger, ABE-related manipulations further enhanced recollection.

## Introduction

With the rapid advancement of technology and the accelerating pace of daily life, we are increasingly required to engage in multitasking. Given the limited capacity of attentional resources, allocating more attention to one task typically impairs performance on concurrent tasks [17]. However, the discovery of the *attentional boost effect* (ABE) challenged this traditional view [47]. In a typical ABE paradigm, participants saw a sequence of background images while simultaneously monitoring superimposed squares for target detection. They needed to memorize the images and while pressing a key to for target squares and ignoring distractors. Typically, compared to rejecting distractor, detecting and responding to a target requires more cognitive resources, which should impair memory for concurrent stimuli. However, in subsequent memory tests, participants are more likely to remember images presented with targets than those presented with distractors. Subsequently, the researchers labeled this effect as the ABE [47] and found that the effect occurs with different types of memory materials (e.g., verbal materials: [31, 42, 44], visual materials: [47, 48]), various memory types (e.g., explicit memory: [4, 26, 45], implicit memory: [43], source memory: [25, 32, 50], context memory: [42, 44]), and is not limited to intentional encoding [3]. Given the counterintuitive nature of the ABE, it is crucial to conduct a comprehensive investigation of this effect to further our understanding of the underlying mechanisms.

Based on a series of behavioral and neuroimaging evidence, researchers have proposed the dual-task interaction model (DTI) to explain the ABE (for a review, see [46]. According to the DTI, during the encoding phase, background items and detection stimuli compete for limited resources, leading to dual-task interference. However, the detection of the target triggers a temporal selective attention mechanism, accompanied by phase activity in the locus coeruleus-norepinephrine (LC-NE, [49, 56], which allows individuals to allocate more attentional resources, ultimately enhancing their processing efficiency for background information presented concurrently with the target.

Although the DTI provides a detailed elucidation of the mechanism underlying the ABE, a persistent controversy remains in the field: whether the ABE is limited to item-specific processing or whether it can simultaneously enhance both item-specific and relational processing. According to the framework of Hunt and Einstein [15], external information can be divided into two subtypes: item-specific information and relational information. The former refers to features that are unique to a separate stimulus, while the latter refers to features that are shared among different stimuli. These two types of information have distinct effects on memory retrieval [16]. Relational information helps individuals delimit a set of potential responses, while item-specific information aids in distinguishing between different responses. Based on the differences in how these two types of information influence memory and attention, we aim to further investigate whether target detection has differential effects on the processing of these two types of information, in order to advance our understanding of the generality of the ABE.

On the one hand, some studies have suggested a distinction in the effects of the ABE-related manipulation on item-specific versus relational information. Building on the ABE paradigm, Spataro et al. [41] initially modified the test phase by employing a Category-Cued Recall Task (CCRT) and a Category Exemplar Generation Task (CEGT) in order to clarify the mechanisms through which the ABE affects abstract lexical representations. Because the discrimination involved in the CCRT primarily relies on item-specific information, whereas those involved in the CEGT relies on relational information [27, 28], a robust ABE should have been observed in both tasks if target detection exerted a positive effect on both types of information. However, their results showed that target detection enhanced performance in the CCRT but not in the CEGT, indicating a dissociation in the effects of the ABE-related manipulation on item-specific and relational information. Subsequently, Meng et al. [26] adjusted the participants’ learning strategy during the study phase, which biased encoding toward item-specific or relational information. They reported that, compared with focusing on item-specific information, when participants focused on relational information, the ABE was dramatically reduced or even eliminated. Recent evidence from the multiple recall paradigm supports the view that the ABE enhanced the encoding of item-specific information but had no effect on the encoding of relational information. Based on converging evidence from distinct perspectives, researchers have proposed an item-specific account of the ABE, which emphasizes that target detection enhances item-specific processing, while having a null impact on relational processing between items [26, 41, 42, 44].

However, the validity of the item-specific framework of the ABE has been challenged in recent years, because several studies have reported that target detection exerts a positive effect on source memory and context memory [3, 25, 32, 42, 44, 50]; While some studies did not find this effect, see [4, 29]. For example, Mulligan et al. [32] systematically examined the impact of target detection on verbal source memory, which involves the relational information between the memory items and the detection stimuli. The results showed a robust ABE in source memory, and this finding has been further confirmed by studies employing visual stimuli as memory materials [3, 50]. In addition, Spataro et al. [42, 44] found that target detection can enhance context memory, which involves relational information between the memory item and its perceptual features. Given that both source memory and context memory examine relational memory from different perspectives [6], these results seem to suggest that the ABE is not limited to item-specific processing, but also affects relational processing.

Given the ongoing debate regarding whether the ABE-related manipulation enhance both item-specific and relational information, we decided to employ an associative recognition paradigm, which relies on participants’ use of inter-item relational information, to examine this issue in detail. In the study phase of associative recognition paradigm [14], memory items are typically presented in pairs (e.g., A-B, C-D, E-F, G-H). During the test phase, testing items are presented either as intact pairs (presented in the same way as in the study phase, e.g., A-B, C-D) or rearranged pairs (where the pairing between two items from the study phase is restructured, e.g., E-H, G-F). Participants are required to make old (intact) or rearranged judgments for the presented item pairs (e.g. [20, 22, 36]). Therefore, successful associative recognition relies on participants' relational processing between different memory items [1].

In addition, prior research often manipulates the semantic relatedness between memory items by using compound pairs (vs. unrelated pairs) as the memory items in the associative recognition paradigm (e.g. [1, 10, 22]). Compound pairs refer to two independent words combined to form a meaningful and semantically coherent word (e.g., combining 'traffic' and 'rules' to form 'traffic rules'); whereas unrelated pairs refer to two words that do not form a meaningful and semantically coherent word (e.g., combining 'apple' and 'hat' to form 'apple hat'). When individuals encode and process compound pairs, they are influenced by pre-existing knowledge, allowing them to process the two words as a whole through top-down processing, without requiring additional cognitive resources [21]. Therefore, individuals are more likely to perceive and encode compound pairs as a single item for processing. In contrast, when individuals encode and process unrelated pairs, due to the lack of semantic relation between the two words, they treat them as two separate items [1, 38]. Therefore, individuals need to engage in additional processing of the relational information between the two unrelated words to complete recognition. Based on the above discussion, we propose that using compound pairs and unrelated pairs as memory materials can further help clarify whether the ABE can simultaneously affect both item-specific processing and relational processing. Specifically, according to the framework of the item-specific account [26, 41, 42, 44], the ABE only affects item-specific processing, then for compound pairs, because individuals can process the two words as a whole without requiring additional relational processing, a significant ABE should be observed (i.e., target pairs should show better associative recognition performance than distractor pairs). For unrelated word pairs, however, since individuals need to rely more on the relational information between the two words, and target detection has a null impact on relational processing, no ABE should be observed. Of course, if target detection enhances both types of information, ABE is expected to be observed in both types of word pairs.

Besides investigating whether the ABE can enhance both types of information, the current study also aims to further explore the neural mechanisms of the effects of target detection on these two types of information. According to the dual-process model, memory recognition can be distinguished into two discrete components: familiarity and recollection [57, 59]. The former is a relatively fast and automatic process, reflecting an individual's sense of familiarity with the memory material; the latter is a more elaborate process, reflecting an individual's detailed retrieval of information from the memory material. Prior studies utilizing the "remember/know" task or the Dual-Process Signal Detection equation have demonstrated that the ABE-related manipulation during encoding leads to higher levels of familiarity [4], and potentially even recollection [3, 19, 24], in recognition tests. However, considering that these findings are limited to the behavioral level and heavily rely on participants' subjective reports, it is difficult to provide relatively direct and comprehensive insights into the cognitive mechanisms of the ABE.

Therefore, we will utilize electroencephalography (EEG), which has high temporal precision, to more directly examine how target detection during encoding affects memory retrieval processing at the neural level. In the field of event-related potentials (ERP), researchers quantify familiarity and recollection by examining two difference waves during associative recognition (i.e., the difference in mean amplitude elicited by intact pairs versus that elicited by rearranged pairs; e.g. [20, 61]). The FN400 effect, occurring in the early time window and primarily distributed over the frontal region, is associated with familiarity, while the LPC effect, occurring in the later time window and mainly distributed over the parietal region, is related to recollection [7, 8, 39]. We focused on these two ERP effects to examine the effects of target detection on associative familiarity and recollection. Besides ERP, time–frequency representations (TFRs) can also provide further insights into the neural mechanisms underlying memory retrieval (for a review, see [33]. In particular, α activity is thought to index the reactivation of mnemonic representations of the stimuli in word memory task [5]. Additionally, converging evidence suggests that γ activity plays a critical role in memory retrieval [11], with early γ activity reflecting associative processing and being associated with familiarity [12]. Thus, time–frequency analysis (TFA) was further conducted to provide a more informative and detailed complement to the ERP findings.

In summary, this study combines the associative recognition paradigm with the ABE paradigm, using compound pairs and unrelated pairs as background items to manipulate individuals' reliance on item-specific and relational information, with the aim of examining whether the ABE can affect both types of information. According to the framework of the item-specific account, if target detection only facilitates item-specific processing, we predict the following dissociative pattern in the results: (a) For compound pairs, since individuals can treat them as a holistic representation with less reliance on relational information, target conditions will show better memory performance than distractor conditions, and this advantage will also be reflected in the corresponding electrophysiological indicators (e.g., FN400 effect, LPC effect and corresponding neural oscillations). (b) For unrelated pairs, since individuals need to rely on additional relational information to encode, the target and distractor conditions should exhibit equivalent associative memory performance, with no significant neural differences. In contrast, if target detection facilitates both item-specific and relational processing, target conditions should show better associative memory performance than distractor conditions for both types of word pairs, and this advantage will also be reflected in the neural indicators.

## Methods

### Participate

A total of 44 undergraduate and graduate students from a local university participated in the experiment. All participants were right-handed, had normal or corrected-to-normal vision, reported no red–green color blindness, and were native Chinese speakers. Nine participants were excluded from subsequent analysis due to interruptions in EEG signal recording (8 participants) or misunderstandings of the instructions (1 participant). Data from the remaining 35 participants (27 females; M_age_ = 20.55 years, SD = 2.23) were included in the final analyses. The study was approved by the Ethics Committee of the School of Psychology at the university. Written informed consent was obtained from all participants prior to participation, and each received 50 RMB as compensation upon completion of the experiment.

In a recent study using verbal materials, Spataro et al. [42, 44], Exp. 1) reported that the effect size associated with the ABE in a context memory task was Cohen’s *d* = 0.68 (corresponding to *f* = 0.55). We used G*Power 3.1 for a 2 (detection stimulus type: target vs. distractor) × 2 (word pair: compound vs. unrelated) repeated-measures ANOVA (α = 0.05, N = 35, and a low correlation among repeated measures r = 0.20) to calculate the post hoc power to detect an ABE, which was 0.99.

### Materials

The materials were selected from "The Top 8000 Most Frequent Words" in the Modern Chinese Frequency Dictionary (1986). To ensure the timeliness of the materials, we incorporated data from the National Language Commission’s Modern Chinese Corpus (www.cncorpus.zhonghuayuwen.org) and excluded words with a frequency more than three standard deviations above the mean. A total of 816 neutral high-frequency words were selected (word frequency ranging from 0.05%—2.89%). These words were paired based on whether they could form a meaningful compound word, resulting in 204 compound pairs and 204 unrelated pairs. Of these, 192 compound pairs and 192 unrelated pairs were served as critical pairs during the study phase, and the remaining 12 compound pairs and 12 unrelated pairs were used as practice items.

To ensure the validity of the study materials, 12 participants who did not take part in the formal experiment rated the critical pairs from the encoding phase on degree of semantic unitization, familiarity, and pleasantness using a 7-point scale (1 = very low unitization/very unfamiliar/very unpleasant, 7 = very high unitization/very familiar/very pleasant). The results showed a significant difference in degree of semantic unitization between the two types of word pairs, *t*(11) = 16.73, *p* < 0.001, Cohen’s *d* = 4.83, with compound pairs rated (M = 6.49, SD = 0.47) significantly higher than unrelated pairs (M = 1.56, SD = 0.72). By contrast, no significant differences were found for familiarity, *t*(11) = 1.08, *p* = 0.304, *BF*_*10*_ = 0.47 (M_compound pairs_ = 5.65, SD = 1.13; M_unrelated pairs_ = 5.74, SD = 1.18), or for pleasantness, *t*(11) = 1.06, *p* = 0.310, *BF*_*10*_ = 0.46 (M_compound pairs_ = 4.33, SD = 0.60; M_unrelated pairs_ = 4.29, SD = 0.66). Subsequently, the critical compound pairs and critical unrelated pairs were evenly distributed, with half paired with red squares and the other half paired with green squares.

To ensure congruence in the degree of semantic unitization between the study and test phases [22], 96 compound pairs were selected from the critical pairs and recombined to create 96 compound rearranged pairs (e.g., “traffic–rules” and “exam–order” recombined as “traffic–order” and “exam–rules,” with word positions preserved). Similarly, 96 unrelated pairs were selected and recombined to create 96 unrelated rearranged pairs. These were then mixed with the remaining 96 compound intact pairs and 96 unrelated intact pairs and presented in randomized order during the testing phase. Similar to the study phase, the four types of test pairs were rated on degree of semantic unitization, familiarity, and pleasantness. The results showed a significant effect of pair type on degree of semantic unitization, *F*(3, 33) = 301.00, *p* < 0.001, *η2 p* = 0.97. Compound intact pairs (M = 6.47, SD = 0.45) and compound rearranged pairs (M = 6.36, SD = 0.57) were rated significantly higher in the degree of semantic unitization than unrelated intact pairs (M = 1.61, SD = 0.74) and unrelated rearranged pairs (M = 1.57, SD = 0.58, all *ps* < 0.001). However, no significant difference was found between the two compound conditions (*p* = 0.561) or between the two unrelated conditions (*p* = 1.000). By contrast, no significant effects were found for familiarity, *F*(3, 33) = 0.34, *p* = 0.796, *BF*_*10*_ = 0.15 (M_compound intact pairs_ = 5.31, SD = 1.12; M_compound rearranged pairs_ = 5.70, SD = 1.04; M_unrelated intact pairs_ = 5.74, SD = 1.19; M_unrelated rearranged pairs_ = 5.73, SD = 1.20), or for pleasantness, *F*(3, 33) = 0.79, *p* = 0.508, *BF*_*10*_ = 0.23 (M_compound intact pairs_ = 4.32, SD = 0.60; M_compound rearranged pairs_ = 4.32, SD = 0.61; M_unrelated intact pairs_ = 4.28, SD = 0.66; M_unrelated rearranged pairs_ = 4.29, SD = 0.65). These results indicate that the materials used in both the study and test phases were appropriately selected.

The detection stimuli consisted of red (RGB: 255, 0, 0) and green (RGB: 0, 255, 0) squares. The color assignment of target and distractor was counterbalanced across participants (Half of the participants were instructed to press a key for red squares, while the other half were instructed to press a key for green squares).

### Procedure

**Fig. 1:**
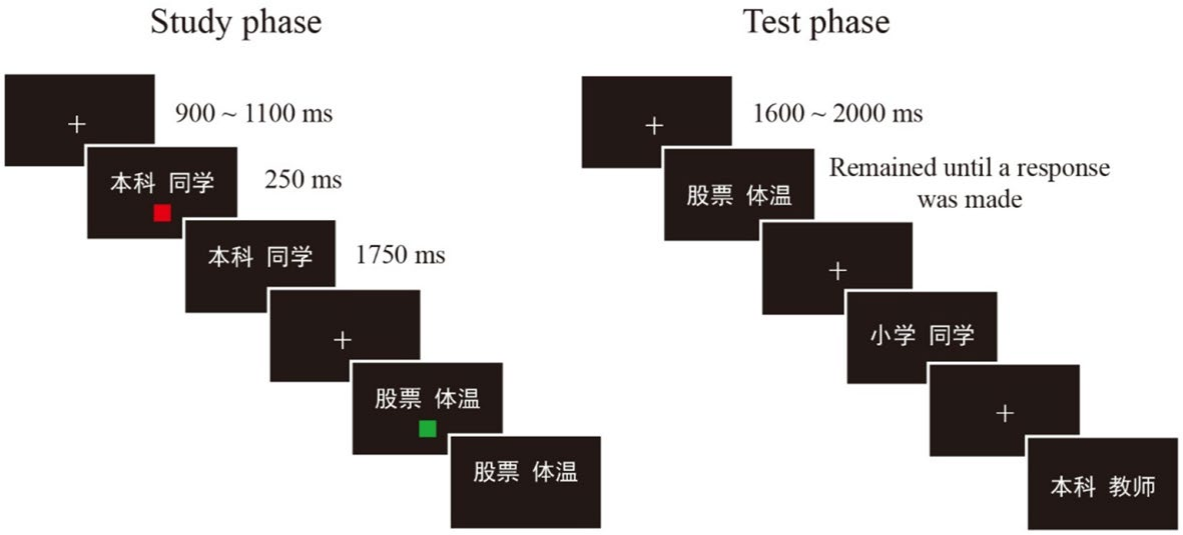
Experimental procedure of the study and test phases in each block of the present study

The experimental procedure is illustrated in Fig. 1. All participants were finished individually in a soundproof room, seated 50 cm from the monitor (DELL Dimension 8200, 15-inch, resolution 1600 × 900). Before the formal experiment, participants completed a practice task to ensure familiarity with the procedure. Given the difficulty of memorizing word pairs, the formal experiment was divided into eight separate blocks, each consisting of a study phase, a distractor task, and a test phase. The following description outlines the procedure for one block:

#### Study phase

Participants were instructed to memorize the word pairs (48) presented on the screen while performing a target detection task. They pressed the space bar whenever a target square appeared and ignored distractor squares. Each trial began with a central fixation cross presented for 900—1100 ms (randomizing the presentation time of the fixation was done to prevent participants from anticipating the appearance of the stimuli). This was followed by the simultaneous presentation of a word pair and a square stimulus, displayed 1 cm above and below the center of the screen, respectively. The pair and square appeared together for 250 ms, after which the square disappeared while the word pair remained on the screen for an additional 1750 ms. Each block contained four types of word pairs: compound target pairs, compound distractor pairs, unrelated target pairs, and unrelated distractor pairs (12 pairs each). All stimuli were presented in a pseudorandom order, with the constraint that no more than three pairs of the same type appeared consecutively.

#### Distractor phase

Participants performed approximately 2 min of continuous two-digit addition and subtraction problems to prevent rehearsal or recall of the word pairs from the study phase.

#### Test phase

Participants were asked to make intact/rearranged judgments on the word pairs (48) presented at the center of the screen. They pressed the “F” key if the pair was judged to be intact and the “J” key if judged to be rearranged. Each trial began with a central fixation cross presented for 1600—2000 ms, followed by the presentation of a word pair at the center of the screen, which remained until a response was made. Each block consisted of 24 intact pairs (six compound–target, six compound–distractor, six unrelated–target, and six unrelated–distractor pairs) and 24 rearranged pairs (six compound–target, six compound–distractor, six unrelated–target, and six unrelated–distractor pairs). All test pairs were presented in a pseudorandom order, with the constraint that no more than three pairs of the same type appeared consecutively.

All stimuli were presented and behavioral data were recorded using E-Prime 2.0. Word pairs were displayed in white, 48-point boldface font. Participants were allowed to rest between blocks until they reported readiness to begin the next block, in order to minimize fatigue.

### EEG recording and preprocessing

The EEG data were recorded using the Neuroscan-64 EEG acquisition and analysis system, with electrode impedance maintained below 5 kΩ. Raw EEG data were amplified using a bandpass filter of 0.05—100 Hz and sampled at 1000 Hz. During online acquisition, participants wore a 64-channel electrode cap following the extended 10—20 international system. The vertex was used as the online reference, and the midpoint between FPZ and FZ served as the ground electrode. Additionally, electrodes were placed at the lateral side of both eyes to record horizontal electrooculogram (HEOG), while electrodes were positioned above and below the left eye to record vertical electrooculogram (VEOG). Before the formal experiment, all participants were instructed to minimize eye movements and body movements during the recording.

EEG data preprocessing was conducted using the EEGLAB toolbox [9] and in-house scripts in Matlab 2018b. First, EEG data were re-referenced to the average of the left and right mastoids (M1, M2), and band-pass filtered from 0.1 to 100 Hz with a notch filter applied at 49—51 Hz. Independent component analysis (ICA) was used to identify and correct artifacts related to eye movements. Continuous EEG signals were segmented into 3—s epochs (− 1000 to 2000 ms relative to stimulus onset). Trials with errors or amplitudes exceeding ± 100 μV were rejected. The mean number of valid and correct trials retained in the test phase for each condition was as follows: compound-target intact pairs (M = 41.83, SD = 5.19), compound-distractor intact pairs (M = 39.11, SD = 6.19), unrelated-target intact pairs (M = 36.31, SD = 6.55), unrelated-distractor intact pairs (M = 34.86, SD = 8.06), compound-target rearranged pairs (M = 29.49, SD = 6.91), compound-distractor rearranged pairs (M = 30.37, SD = 7.43), unrelated-target rearranged pairs (M = 39.37, SD = 5.93), and unrelated-distractor rearranged pairs (M = 40.14, SD = 5.88).

For ERP analyses, the data were re-filtered with a 0.1—30 Hz band-pass filter and segmented into 1.2—s epochs (− 200 to 1000 ms relative to stimulus onset). The data before the stimulus (− 200—0 ms) is used for baseline correction. The remaining correct trials in each condition were then averaged. For TFA, the sampling rate was first down-sampled to 500 Hz (in order to reduce computational load), and EEG data were segmented into 2.3—s epochs (− 800 to 1500 ms relative to stimulus onset).

### Data analysis

#### Behavioral data

For the target detection task during the study phase, we report participants’ hit rates and reaction times (RTs) to target stimuli, as well as false alarm rates to distractor stimuli, to ensure that they performed the task attentively. Paired-sample *t*-tests were then conducted on hit rates and RTs for target stimuli paired with the two word pair conditions.

For the associative recognition test, we focused on three measures: the hit rate for intact pairs (i.e., the proportion of intact pairs correctly judged as “intact”), the false alarm rate for rearranged pairs (i.e., the proportion of rearranged pairs incorrectly judged as “intact”), and the performance of recognition (Pr, calculated as hit rate minus false alarm rate, which is commonly used as an index of associative memory, e.g. [1, 20, 21]).^1^ These measures were analyzed using a 2 (detection stimuli type: target vs. distractor) × 2 (word pair: compound vs. unrelated) repeated-measures ANOVA.

#### ERP analyses

Based on the definitions of the FN400 and LPC effects, together with inspection of the waveforms and topographical maps, the FN400 effect was quantified as the difference in mean amplitude between correctly identified intact pairs (hits) and correctly identified rearranged pairs (correct rejections) within the 350–550 ms time window at three frontal electrodes (F3, Fz, F4). The LPC effect was quantified as the difference in mean amplitude between correctly identified intact pairs and correctly identified rearranged pairs within the 550—750 ms at three parietal electrodes (P3, Pz, P4). Then, to determine whether significant differential components exist under each condition, we will perform one-tailed, one-sample *t*-tests to assess whether the magnitude of the FN400 and LPC effects is significantly greater than zero under each condition separately. Finally, the mean amplitudes of the FN400 and LPC effects were submitted to 2 (detection stimulus type: target vs. distractor) × 2 (word pair: compound vs. unrelated) repeated-measures ANOVAs respectively.

#### TFA

The short-time Fourier transform with a Hanning window of 200 ms was then applied to estimate the TFR of each trial in the 5—100 Hz range. The spectral power was averaged across trials for each participant, and event-related spectral perturbation (ERSP) values were computed using a baseline of − 500 to—100 ms relative to stimulus onset, expressed in decibels (dB). To provide a more comprehensive characterization of the present study, subsequent analyses focused on event-related spectral perturbations (ERSPs) within the 0–1000 ms time window. The time–frequency regions of interest (TF-ROIs) and spatial regions of interest (sROIs) were defined in an exploratory and data-driven manner.

**Fig. 2:**
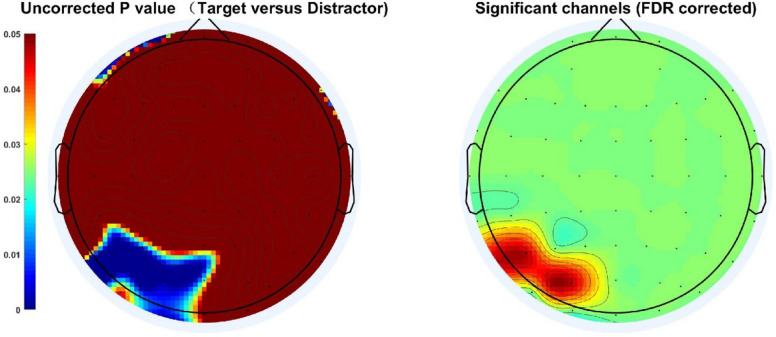
Topographic maps showing the main effect of detection stimuli type in the TF-ROI (50 ~ 60 Hz, 400 ~ 500 ms). **A** Topographic map of the uncorrected *p*-values for the main effect of detection stimuli type (The color bar represents *p*-values, with colors closer to blue indicating *p*-values closer to 0). **B** Channel distribution of the main effect of detection stimuli type after FDR correction (with red regions indicating significant channels: P7, P5, PO5, PO3)

First, inspection of the TFRs at several representative electrodes revealed three ERSP changes relative to baseline: (1) γ event-related synchronization (ERS, 35—60 Hz) in the 150—250 ms, (2) γ ERS (50—60 Hz) in the 400—500 ms, and (3) α event-related desynchronization (ERD, 9—13 Hz) in the 400—1000 ms. These three time–frequency ranges were selected to perform pairwise comparisons between all conditions for intact pairs across all channels. In order to control for false positives, *p*-values were corrected using the false discovery rate (FDR) method. The results showed a significant difference in the 400–500 ms γ ERS only between target intact pairs and distractor intact pairs, with significant electrodes mainly distributed over the left parietal—occipital region (P7, P5, PO5, PO3, *ps* < 0.05, See Fig. 2). No other significant effects were observed at any electrode (*ps* > 0.05). Therefore, the γ ERS (50—60 Hz, 400—500 ms) was defined as the TF-ROI, and the left parietal region (P7, P5, PO5, PO3) as the sROI. Finally, the mean power within the TF-ROI across the left parietal–occipital electrodes (P7, P5, PO5, PO3) was computed for each condition ([P7 + P5 + PO5 + PO3]/4) and submitted to a 2 (detection stimuli type: target vs. distractor) × 2 (word pair: compound vs. unrelated) repeated-measures ANOVA.

When the sphericity assumption was violated, the Greenhouse–Geisser correction was applied. For multiple comparisons, *p*-values were adjusted using the Bonferroni method. In addition, given the limitations of null hypothesis significance testing (NHST) in evaluating the null hypothesis, we reported Bayes factor (*BF*_*10*_) for non-significant results to provide more comprehensive evidence. A BF10 between 1 and 3 indicates weak evidence for H1; a *BF*_*10*_ between 1/3 and 1 indicates weak evidence for H_0_; a *BF*_*10*_ between 1/10 and 1/3 indicates moderate evidence for H_0_; and a *BF*_*10*_ less than 1/10 indicates strong evidence for H_0_ [52, 53]. All analyses were conducted using Jamovi 2.3.28.

## Results

### Behavior performance

#### Target detection task

The mean hit rate for targets paired with compound pairs was 97.47% (SD = 4.62), and the mean hit rate for targets paired with unrelated pairs was 96.96% (SD = 4.08). The mean false alarm rate for distractors paired with compound pairs was 1.13% (SD = 1.50), and the mean false alarm rate for distractors paired with unrelated pairs was 0.95% (SD = 1.02). These results suggest that the participants completed the target detection task attentively. The difference in hit rates between the two word pair conditions was not significant, *t*(34) = 1.15, *p* = 0.259, *BF*_*10*_ = 0.33. The difference in false alarm rates between the two word pair conditions was also not significant,* t*(34) = 0.70, *p* = 0.488, *BF*_*10*_ = 0.23. However, a significant difference was found in RTs, *t*(34) = 2.14, *p* = 0.039, Cohen’s *d* = 0.36. Participants responded faster to targets paired with compound pairs (M = 810.52 ms, SD = 181.23) than to those paired with unrelated pairs (M = 823.73 ms, SD = 195.03).

#### Associative recognition task

Participants’ hit rates for intact pairs, false alarm rates for rearranged pairs, and Pr in each condition are shown in Table 1. For hit rates, repeated-measures ANOVA revealed a significant main effect of detection stimuli type, *F*(1, 34) = 17.08, *p* < 0.001, *η2 p* = 0.33, with higher hit rates for target-intact pairs than for distractor-intact pairs. The main effect of word pair was also significant, *F*(1, 34) = 25.02, *p* < 0.001, *η2 p* = 0.42, with higher hit rates for compound-intact pairs than for unrelated-intact pairs. The interaction between detection stimuli type and word pair was not significant, *F*(1, 34) = 2.05, *p* = 0.161, *BF*_*10*_ = 0.36.

**Table 1:** Mean (SD) of hit rate, false alarm rate, and Pr in associative recognition for each condition

Condition	Compound Pairs		Unrelated Pairs	
	**Target**	**Distractor**	**Target**	**Distractor**
Hit Rate	0.89 (0.10)	0.83 (0.13)	0.77 (0.13)	0.74 (0.17)
False alarm rate	0.38 (0.17)	0.36 (0.15)	0.17 (0.12)	0.14 (0.11)
Pr	0.51 (0.17)	0.47 (0.20)	0.61 (0.22)	0.60 (0.21)

For false alarm rates, repeated-measures ANOVA revealed a significant main effect of detection stimuli type, *F*(1, 34) = 5.80, *p* = 0.022, *η2 p* = 0.15, with higher false alarm rates for target-rearranged pairs than for distractor-rearranged pairs. The main effect of word pair was also significant, *F*(1, 34) = 95.71, *p* < 0.001, *η2 p* = 0.74, with higher false alarm rates for compound-rearranged pairs than for unrelated-rearranged pairs. The interaction between detection stimuli type and word pair was not significant, *F*(1, 34) = 0.12, *p* = 0.728, *BF*_*10*_ = 0.24.

For Pr, repeated-measures ANOVA revealed a significant main effect of detection stimuli type, *F*(1, 34) = 4.31, *p* = 0.045, *η2 p* = 0.11, with higher Pr for target pairs than for distractor pairs. The main effect of word pair was also significant, *F*(1, 34) = 30.21, *p* < 0.001, *η2 p* = 0.47, with higher Pr for unrelated pairs than for compound pairs. The interaction between detection stimuli type and word pair was not significant, *F*(1, 34) = 1.48, *p* = 0.232, *BF*_*10*_ = 0.42.

The behavioral data revealed the following main findings: (1) Participants exhibited better associative memory for target pairs compared to distractor pairs, demonstrating a clear ABE. (2) No significant interaction was found between detection stimulus type and word pair, indicating that the ABE was observed for both compound and unrelated pairs. These findings suggest that target pairs exhibit better associative recognition performance than distractor pairs for both types of word pairs, supporting the hypothesis that target detection can enhance both item-specific and relational information.

### ERP results

**Fig. 3:**
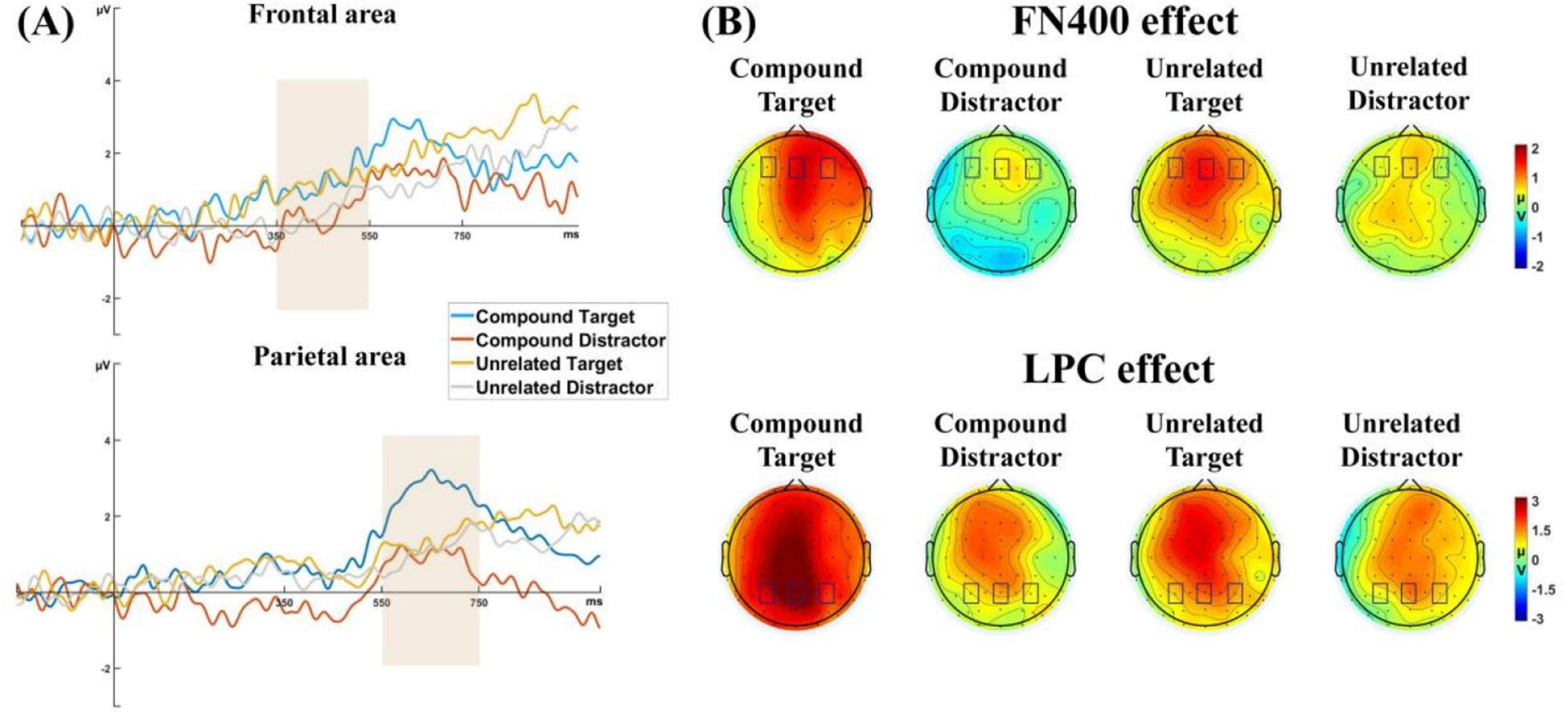
ERP Waveforms and Topographic Maps. **A** Average waveforms of the FN400 effect (frontal region, 350—550 ms) and LPC effect (parietal regions, 550—750 ms) under each condition (blue line: compound-target pairs; red line: compound-distractor pairs; yellow line: unrelated-target pairs; unrelated distractor pairs). The FN400 and LPC effect values were calculated as the difference in average amplitude between correctly identified intact pairs (hits) and correctly identified rearranged pairs (correct rejections). The shaded boxes represent the time windows corresponding to the respective ERP components. **B** Topographic maps of the FN400 and LPC effects under each condition [The displayed values represent the difference in average amplitude between correctly identified intact pairs (hits) and correctly identified rearranged pairs (correct rejections)]

FN400 Effect (350–550 ms, see Fig. 3). The results of the one-sample *t*-tests indicated that the magnitude of the FN400 effect was significantly greater than zero for both compound-target pairs [*t*(34) = 3.83, *p* < 0.001, Cohen’s d = 0.65] and unrelated-target pairs [*t*(34) = 3.20, *p* = 0.003, Cohen’s d = 0.54], evoking a robust FN400 effect. In contrast, the FN400 effect was not significantly greater than zero for either compound-distractor pairs [*t*(34) = 0.79, *p* = 0.436, *BF*_*10*_ = 0.38] or unrelated-distractor pairs [*t*(34) = 1.06, *p* = 0.295, *BF*_*10*_ = 0.52], with no significant FN400 effect evoked.

Repeated-measures ANOVA revealed a significant main effect of detection stimulus type, *F*(1, 34) = 6.57, *p* = 0.015, *η2 p* = 0.16, indicating that target pairs elicited greater familiarity processing than distractor pairs. The main effect of word pair was not significant,* F*(1, 34) = 0.01, *p* = 0.924, *BF*_*10*_ = 0.18, indicating that familiarity did not differ between the two types of word pairs. The interaction between detection stimulus type and word pair was also not significant,* F*(1, 34) = 0.03, *p* = 0.871, *BF*_*10*_ = 0.26, indicating that the influence of target detection on the familiarity did not differ between the two types of word pairs.

LPC Effect (550–750 ms, see Fig. 3). The results of the one-sample* t*-tests revealed that the magnitude of the LPC effect was significantly greater than zero across all conditions [compound-target pairs:* t*(34) = 5.92, *p* < 0.001, Cohen’s d = 1.00; compound-distractor pairs: *t*(34) = 3.25, *p* = 0.001, Cohen’s d = 0.55; unrelated-target pairs: *t*(34) = 4.60, *p* < 0.001, Cohen’s d = 0.78; unrelated-distractor pairs: *t*(34) = 3.19, *p* = 0.002, Cohen’s d = 0.54], indicating that a significant LPC effect was evoked in all conditions.

Repeated-measures ANOVA revealed a significant main effect of detection stimulus type, *F*(1, 34) = 11.12, *p* = 0.002, *η2 p* = 0.25, indicating that target pairs elicited greater recollection processing than distractor pairs. The main effect of word pair was not significant,* F*(1, 34) = 3.20, *p* = 0.083, *BF*_*10*_ = 0.68, indicating that recollection did not differ between the two types of word pairs. However, the interaction between detection stimuli type and word pair was marginally significant, *F*(1, 34) = 4.06, *p* = 0.052, *η2 p* = 0.11, *BF*_*10*_ = 2.45.

Simple effects analysis revealed that (1) for compound pairs, the magnitude of the LPC effect was significantly greater in the target condition than in the distractor condition (*p* = 0.005); for unrelated pairs, the magnitude of the LPC effect did not differ significantly between the target and distractor conditions (*p* = 1.000). (2) For the target condition, the magnitude of the LPC effect for compound pairs did not differ significantly from that for unrelated pairs (*p* = 0.113); for the distractor condition, the magnitude of the LPC effect for compound pairs did not differ significantly from that for unrelated pairs (*p* = 1.000).

Therefore, the results suggest the following: (1) Although participants exhibited significant LPC effects for all word pairs, the magnitude of the LPC effect was larger in the target condition compared to the distractor condition; (2) Compared to unrelated pairs, target detection tended to enhance recollection more strongly for compound pairs numerically. However, given that the interaction between the two factors was marginally significant and the Bayesian factor analysis provided only weak evidence, this pattern remains tentative and only reflects a trend of difference.

### TFA results

**Fig. 4:**
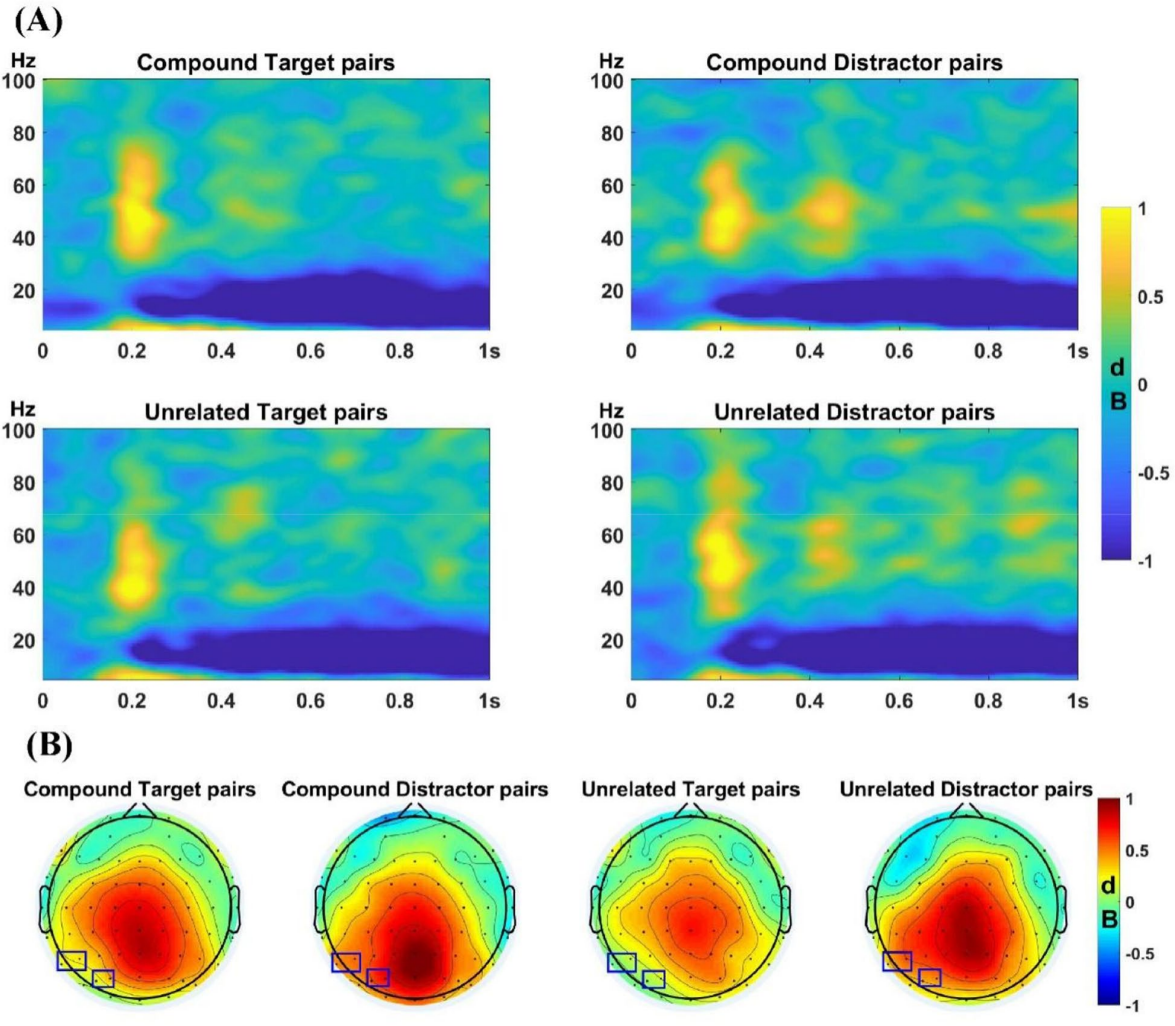
Results of the TFA. **A** Time–frequency representations obtained by averaging across the four channels in the left parieto-occipital region (P7, P5, PO5, PO3) under each condition. **B** Topographic maps of γ-band activity (50—60 Hz, 400—500 ms) under each condition (the blue rectangle indicates the s-ROI in the TFA)

γ-ERS (50—60 Hz, 400—500 ms, see Fig. 4). Repeated-measures ANOVA revealed a significant main effect of detection stimuli type, *F*(1, 34) = 17.11, *p* < 0.001, *η2 p* = 0.34, with lower power for target pairs than for distractor pairs. The main effect of word type was not significant, *F*(1, 34) = 2.27, *p* = 0.141, *BF*_*10*_ = 0.64. The interaction between detection stimuli type and word type was also not significant, *F*(1, 34) = 0.13, *p* = 0.724, *BF*_*10*_ = 0.26.

## General discussion

The current study combines the associative recognition paradigm with the ABE paradigm, using compound pairs and unrelated pairs as memory items to manipulate the reliance on relational information between items, in order to investigate the effects of ABE-related manipulation on item-specific vs. relational information and its underlying neural mechanisms. The behavioral results showed that (a) word pairs presented with target were recognized more accurately in the subsequent associative recognition test than those presented with distractor, and this advantage was not influenced by word pair type. The EEG results further provided neural-level evidence for the behavioral findings. The ERP results revealed that (b) target pairs evoked a significant FN400 effect, which reflects familiarity-based processing, whereas distractor pairs did not evoke this effect. (c) Additionally, the effect of the ABE-related manipulation on the LPC component, which reflects recollection-based processing, is dissociated across different types of word pairs. Specifically, only under the compound pair condition did the target pairs elicit a larger LPC effect compared to the distractor pairs; under the unrelated pair condition, there was no difference in LPC effect between the target and distractor pairs. Finally, the TFA results showed (d) that target pairs induced lower γ-band power, which is linked to familiarity, than distractor pairs.

According to the item-specific account of the ABE, target detection is assumed to enhance item-specific processing but not relational processing between items [26, 41, 42, 44]. On this view, the ABE should be observed for compound pairs, which can be processed as single units, but not for unrelated pairs, which require additional relational processing for successful encoding [1, 21, 22, 38]. However, inconsistent with the predictions of this framework, we found that the ABE-related manipulation facilitated participants' associative memory under both compound pairs and unrelated pairs, indicating that target detection can also enhance relational processing. Our point of interest is why some previous studies failed to observe the ABE in relational processing. We speculate that this may be due to discrepancies between the experimental paradigms. In the series of studies mentioned in the introduction [26, 41, 42, 44], memory items were presented in a single and isolated form during both studying and testing. Therefore, relational processing of the items may be considered inter-trial association (see [42, 44]). In contrast, in the present study, the two items within each word pair were presented simultaneously, yielding within-trial relational processing. Turker and Swallow [50] proposed that LC-NE activity leads to an increase in long-term potentiation in the dentate gyrus of the hippocampus, a structure that plays an important role in the relational encoding of multiple items presented at the same time [40], suggesting that the ABE may be particularly sensitive to within-trial relational information. Furthermore, previous evidence suggests that LC-NE activity triggered by target is transient [2, 35] and selectively enhances the processing of stimuli presented concurrently with the target [48]. Consequently, when the relationship between items involves inter-trial associations, the ABE is attenuated due to the temporally constrained nature of LC-NE activity. In contrast, when the relational information involves within-trial association, the enhancement induced by LC-NE activity is maximized, thereby enabling the ABE to be fully observed.

This framework has also been supported by recent studies. Wang and Egner [54] showed that target detection enhanced item memory for images but had no effect on temporal order and temporal distance memory, both of which require establishing temporal associations between items. Broitman and Swallow [4] found that target detection did not influence temporal context representations in free recall. In these two studies, the representations of background items need to be associated across different time, which may preclude an ABE given the transient nature of LC–NE activity. Therefore, our findings further extend the DTI [46], indicating that target detection can facilitate both item-specific and relational processing, but only when relevant information temporally overlaps with the target.

The EEG results further provide an explanation from the perspective of neural mechanisms for the ABE. In the ERP components related to familiarity, we found that target detection elicited a significant FN400 effect in subsequent associative recognition, whereas distractor rejection did not. In associative recognition, recollection plays a critical role in supporting this discrimination, because participants must accurately remember which items were previously paired together [36]. In contrast, because both intact and rearranged pairs contain studied items, they are assumed to elicit comparable levels of familiarity, rendering familiarity insufficient to support associative recognition [1, 23, 58]. However, we found that target detection during encoding allows familiarity to contribute to associative recognition, suggesting that the ABE-related advantage in associative memory is partly due to target detection enabling individuals to make discrimination based on familiarity.

Then, how does the ABE-related manipulation during encoding influence familiarity-based processing? From the perspective of neural oscillations, it seems that this may provide additional insights. The TFA results showed that target pairs induced lower γ-band activity compared to distractor pairs in the left parietal—occipital region. It is generally believed that gamma-band activity is related to familiarity [12], with activity in the parietal region reflecting the amount of evidence accumulation during memory decision-making [55]. Two studies have found that target detection during the retrieval leads participants to adopt a more lenient criterion, increasing their tendency to judge items as "old" (ABE-related bias, [13, 37]. Huang and Meng [13] speculated that target detection may have led to greater processing fluency for background information, thereby increasing both the hit rate and false alarm rate for the paired items (e.g. [18, 34]). A similar behavioral bias was also observed in our study (particularly the false alarm rate for target rearranged pairs). Thus, we speculate that target detection enhances fluency-based familiarity, rather than item familiarity, enabling individuals to rely on less information during recognition decisions, thereby facilitating associative memory.

Moreover, we found that although significant LPC effects were induced across all conditions, the LPC effect for target pairs was significantly larger than for distractor pairs. This suggests that target detection not only enhanced automatic familiarity processing but also facilitated subsequent elaborative recollection processing. However, this finding seems to be inconsistent with previous research. Mulligan et al. [31] suggested that the ABE occurs only during the initial phases of word identification and comprehension, rather than during the later phases of controlled and elaborative processing [30, 31]. Compared to familiarity, recollection involves more elaborate operations. Therefore, target detection is unlikely to facilitate recollection processing. We speculate that this difference may stem from the varying demands of the experimental task on familiarity and recollection. Mulligan et al. used item recognition paradigm, in which old items elicit higher levels of familiarity than new items, allowing participants to make recognition judgments based solely on familiarity [36, 58, 60]. In contrast, in the associative paradigm, as previously mentioned, participants rely more heavily on recollection. We speculate that in the item recognition paradigm, where familiarity plays a primary role, ABE-related benefits mainly affect early processing. In contrast, in the associative recognition paradigm, where recollection is more important, ABE-related benefits may influence later, elaborative processing. Future research could test this possibility by adding item recognition to the current study.

Interestingly, we also observed a trend: the enhancement of recollection by target detection varied across different word pairs. Specifically, target detection appears to yield greater benefits for recollection of compound pairs compared to unrelated pairs. Relevant evidence suggests that the ABE may operate at different levels for verbal and visual materials. For verbal materials, the ABE primarily enhances encoding at the level of abstract, amodal representations rather than at the perceptual level [30, 31, 45]. This distinction is particularly relevant here, because in the present study both compound and unrelated pairs consisted of two words presented simultaneously in the visual modality, and thus did not differ substantially at the perceptual level. If target detection merely facilitated processing at the perceptual level, its effect on recollection for both types of word pairs should have followed the same pattern. However, we found that when the semantic relationship between the two words in a word pair is strong, target detection tends to yield greater benefits for elaborative processing. We speculate that when encoding unrelated pairs, the lack of semantic relationship between the two words leads participants to form independent lexical representations for each word. As a result, the benefits from ABE-related manipulations are relatively limited. In contrast, when encoding compound pairs, participants are able to form both individual representations for each word and a rich lexical representation for the pair as a whole. This leads to the maximization of benefits from ABE-related manipulations. Nonetheless, we must acknowledge that, due to the relatively limited evidence provided by our data, the observed pattern remains tentative and requires further verification in future studies to confirm its reliability.

Finally, we observed an unexpected but noteworthy finding. Although compound pairs are typically assumed to induce unitization and facilitate associative memory (e.g. [1, 21, 36]), compound pairs were remembered worse than unrelated pairs in the present study, with no corresponding differences in electrophysiological indicators. This suggests that unitization had a null or even negative effect on associative memory in this context. One possible explanation is that the effects of unitization and target detection during encoding may be redundant. Previous studies have shown that unitization enhances associative memory by increasing the contribution of familiarity or recollection [1, 36, 61], whereas target detection in the current study also influenced both processes. Because the target appears within the 0—250 ms time window relative to the word pair presentation, this may cause the ABE to operate during the relatively early stages. However, the unitization manipulation in this study is conceptual unitization [20], and its processing occur during the relatively later stages of conceptual processing. Given the ABE-related manipulation has already sufficiently enhanced encoding processing in the early stages, the effects of unitization occurring later are minimized. Future studies using complementary cognitive neuroscience methods may help further clarify this possibility.

## Conclusion

The current study used the associative recognition paradigm to examine whether ABE-related manipulations can both enhance item-specific and relational processing. We found that target detection enhances associative memory, indicating that ABE can affect relational information. At the neural mechanism level, target detection enhances the contribution of fluency-based familiarity and recollection in associative recognition. When the semantic relationship between items is strong, target detection tends to produce a greater enhancement of recollection. Our findings extend the framework of DTI, deepening our comprehensive understanding of the ABE.

## Data Availability

All datasets, procedures, materials and code used in this study are available upon request from the corresponding author.

## Abbreviations

ABE: Attentional boost effect
DTI: The dual-task interaction model
LC-NE: Locus coeruleus-norepinephrine
CCRT: Category-Cued Recall Task
CEGT: Category Exemplars Generation Task
EEG: Electroencephalography
ERP: Event-related potentials
FN400: Frontal Negativity 400
LPC: Late Positive Component
TFA: Time–frequency analysis
TFR: Time–frequency representations
TF-ROIs: Time–frequency regions of interest
S-ROIs: Spatial regions of interest
ERSP: Event-related spectral perturbation
ERS: Event-related synchronization
ERD: Event-related desynchronization

## Footnotes

1 We should acknowledge that Ahmad and Hockley [1] employed both Pr and the signal detection theory indicator *d′* as dependent variables. However, it is important to note that in the present study, seven participants (accounting for 20% of the total sample) exhibited ceiling or floor perform
